# Characterization and structural analysis of alcohol-fractionated lignin biofuels processed at ambient temperature

**DOI:** 10.1016/j.heliyon.2024.e39249

**Published:** 2024-10-11

**Authors:** Tor I. Simonsen, Saket Kumar, Demi T. Djajadi, Jacob J.K. Kirkensgaard, Jens Risbo, Sune T. Thomsen, Yohanna C. Orozco

**Affiliations:** a Department of Geosciences and Natural Resource Management, University of Copenhagen, Frederiksberg C, Copenhagen, 1958, Denmark; b Department of Chemical Engineering, School of Energy Technology, Pandit Deendayal Energy University, Gandhinagar, 382007, India; c Department of Food Science, University of Copenhagen, Frederiksberg C, Copenhagen, 1958, Denmark; d Niels Bohr Institute, Faculty of Science, University of Copenhagen, Universitetsparken 5, Copenhagen, 2100, Denmark

**Keywords:** Lignin, Fuel, Cold-processed lignin in ethanol oil (CLEO), Alcohol fractionation, Sustainable maritime fuel

## Abstract

This study introduces Cold-processed Lignin in (M)ethanol Oil (CLEO/CLiMO), a novel biofuel technology derived from the alcohol-fractionation of lignin at ambient temperatures, offering a sustainable alternative to conventional marine fuels. The production process achieved solid loadings of up to 60 wt% lignin and a volumetric energy density 39 % higher than pure alcohols. Lignin concentrations above 30 wt% promoted colloidal stability through the proposed formation of a spanning network of lignin aggregates, associated with a 100-fold increase of viscosity. Additionally, we observed a decrease in the radius of gyration of lignin particles from 2.5 to 2.7 nm at 30 wt% to 1.1–1.3 nm at 60 wt% following a transition from globular to elongated random coil shaped particles. This was accompanied by a twofold increase in the partial specific volume of lignin, suggesting a reduction in packing efficiency. The study highlights CLEO's potential as a sustainable shipping fuel alternative, combining favorable fuel properties with a simple and scalable production method.

## Highlights

• A simple lignin fuel - Cold-processed Lignin in (M)ethanol Oil (CLEO/CLiMO).
• Stability was achieved via a lignin aggregate network.
• Biofuel achieved high energy density, up to 27.8 MJ/L.

## 1 Introduction

The maritime industry is facing a transition towards sustainable shipping solutions, where the International Maritime Organization (IMO) targets the reduction of greenhouse gas emissions from the sector by 50 % by 2050 compared to 2008 levels [1]. This significant shift stresses the pressing need for renewable alternatives to conventional marine fuels. In this landscape, lignin, an abundant natural aromatic polymer found in lignocellulosic biomass, stands out as a promising renewable resource with potential for sustainable biofuel production [2,3]. Despite its energy-rich aromatic structure, lignin remains largely underutilized [4,5].

Lignin-first methods represent a shift in biomass valorization strategies with focus on the early fractionation and depolymerization of lignin during biomass processing, which can lead to more efficient valorization of all biomass components, emphasizing lignin extraction for high-value chemical and fuel production [6]. For instance, Reductive Catalytic Fractionation (RCF) transforms lignin residue into crude ‘bio-oil’, rich in low molecular weight aromatics by extracting, depolymerizing and avoiding repolymerization of lignin at high temperatures, using redox-active catalysts like Pd or Ru in a polar protic solvent often supplied by hydrogen [[7], [8], [9]]. However, the high energy requirements and harsh reaction conditions makes the commercial scalability of RCF challenging [[10], [11], [12]].

A milder processing alternative is thermolytic solvolysis, which partially depolymerizes lignin in polar protic solvents at 180–250 °C, producing alcoholic lignin dispersions [13,14]. This method is simpler and less costly than RCF, and has been proposed for maritime fuel applications, given the lower fuel quality requirements of ship engines [[14], [15], [16]]. However, issues such as lignin aggregation and sedimentation present significant obstacles for maintaining storage stability and viscosity. These are crucial factors for shipping fuels in compliance with ISO standards [17].

This study introduces a novel and simple approach: Cold-processed Lignin in Ethanol/Methanol Oil (CLEO/CLiMO), where alcohol fractionated lignin dispersions are stabilized through partial solvent evaporation reaching lignin concentrations between 30 wt% and 60 wt%. Recent findings demonstrated how lignin dispersions from thermal solvolysis could be stabilized by adjusting the lignin concentrations [15,18]. Kumar et al. (2022) suggested the apparent stability was linked to the formation of a network of interacting lignin molecules.

To further understand this stabilization phenomenon and evaluate the potential use of lignin-alcohol fuels as an alternative maritime fuel, this paper strives to reduce the production complexity by circumventing thermal solvolysis and produce stable lignin dispersions at ambient temperature. This is achieved through the analysis of ash content, viscosity, and calorimetry, as well as through structural analysis using Small Angle X-ray Scattering (SAXS), rheology and partial specific volume calculations.

## 2 Materials and methods

### 2.1 Reagents

Commercial Protobind 1000 soda lignin (PB1000) with 96 % dry matter content was obtained from TaNovis, Switzerland. According to the product sheet, PB1000 is characterized by the following properties:

• Number average molecular weight: 750–1000 g/mol
• Weight average molecular weight: 2500–4000 g/mol
• Aliphatic OH content: 1.8–2.1 mmol/g
• Phenolic OH content: 2.7–3.1 mmol/g
• Acid content: 0–1.2 mmol/g

Laboratory-grade ethanol (96 %, v/v) and methanol (100 %) were obtained from VWR International, USA.

### 2.2 Production of lignin biofuel

Ethanol or methanol solutions (90:10 alcohol:water ratio, w/w) were mixed with PB1000 lignin at a 1:5 solid to liquid ratio (w/w) at ambient temperature. The mixture was stirred at 700 RPM for 1 h, and the undispersed lignin was subsequently separated via centrifugation at 1000*g* for 10 min. To calculate yield, 4 × 1.0 g of each fraction were dried in a vacuum oven at 40 °C until constant weight. The fractionation yield, expressed as wt%, was determined by calculating the ratio of dry weight between the dispersed lignin and the unfractionated lignin.

CLEO or CLiMO was produced by evaporating alcohol from the dispersed lignin using a Buchi Rotavapor R300 (water bath at 50 °C, coolant at 10 °C, pressure at 175 mbar for ethanol and 337 mbar for methanol) until lignin concentration reached 60 wt%.

The water content of CLEO and CLiMO was determined to be approximately 10 wt% (±0.5 wt%) using a Mettler Toledo Density2Go portable densitometer.

Nine dilutions of the 60 wt% lignin dispersions were prepared using the 90:10 alcohol:water mixtures to create dispersions with 1–60 wt% lignin.

### 2.3 Density

Density measurements were conducted gravimetrically and performed in triplicates. Samples were transferred using a 1.0 ml Microman E M1000E positive displacement pipette and weighed to an accuracy of ±0.1 mg.

### 2.4 Sample stability

Similar to previously reported [15,19], stability assessment involved refrigerating 10–20 ml of sample at 5 °C for 24 h, followed by reconditioning to ambient temperature and centrifuging at 1000 g for 10 min. Stability was determined based on the absence of precipitation or the presence of a small, miscible smear after vortexing for 5 s. Instability was defined by the presence of an immiscible pellet.

### 2.5 Energy density

Higher heating values were measured on a Parr 6400 Bomb calorimeter, using a method based on ASTM D240 as detailed in Ref. [15].

### 2.6 Ash content analysis

Ash content was measured gravimetrically by adding 5.0 g of oven dry sample in dried ceramic crucibles covered by punctured aluminum foil and heated at 550 °C for 3 h in a muffle furnace. Ceramic crucibles were transferred to a desiccator and weighed after being cooled to ambient temperature.

### 2.7 Elemental analysis

Elemental analysis was done by inductively coupled plasma-mass spectrometry (ICP-MS), which is further detailed in the supplementary materials.

### 2.8 Flow curve measurement

Flow curve experiments were conducted according to previously established protocols [15], with shear rates ranging from 10^−1^ to 10^3^ s^−1^ and an equilibration time set to 1200 s [15].

### 2.9 Flow curve analysis

Shear thinning and constant viscosity regime were modeled using the Sisko model:

$$\eta =\eta_{\infty }+K{\dot{\gamma }}^{n-1}\text{,}$$

where, η is shear viscosity, $\eta_{\infty }$ is high shear viscosity, *K* is fluid consistency index, $\dot{\gamma }$ is shear rate, and *n* is flow behavior index. Newtonian behavior corresponds to *n* = 1, shear thinning to *n* < 1 and shear thickening to *n* > 1.

### 2.10 Small-angle X-ray scattering

SAXS experiments were conducted as per methods described in Kumar et al. (2022). Data fitting employed a combination of a power law and a Gaussian coil model for low and high Q regions, respectively. More details on the fitting parameters are found in supplementary materials, Table S1.

### 2.11 Partial specific volume

Partial specific volume was calculated based on the partial derivative of volume with respect to the mass of the component. Polynomial fitting and bootstrap procedures were employed for statistical assessments, as detailed in supplementary materials.

## 3 Results and discussion

### 3.1 Alcohol fractionation

The initial step in CLEO/CLiMO production is solvent fractionation of lignin. The extraction yields were 48 wt% (±4 %) and 54 wt% (±2 wt%) using ethanol and methanol, respectively. These yields are comparable to those reported for lignin solvolysis oil using the same PB1000 lignin, where two recent studies demonstrated extraction yields ranging from 57 % to 64 % for ethanol and methanol [14,15]. It is noteworthy that the additional yield attained through thermolytic solvolysis was only 7–10 percentage points higher compared to alcohol fractionation at ambient temperature. This gap might be compensated by improving the solvent composition, as previous fractionation studies have shown yield gains by mixing up to 30–40 wt% water in ethanol [[20], [21], [22]]. However, there is a tradeoff between yield and energy density of the resulting fuel, where using 10 wt% water prevented water from accumulating in the CLEO/CLiMO fuel during solvent evaporation [19]. Although this concentration exceeds the recommended ISO8217 value of ≤0.5 % (v/v) [17], the addition of water in fuel can reduce NOx emissions in marine diesel engines [23].

### 3.2 Colloidal stability

**Fig. 1:**
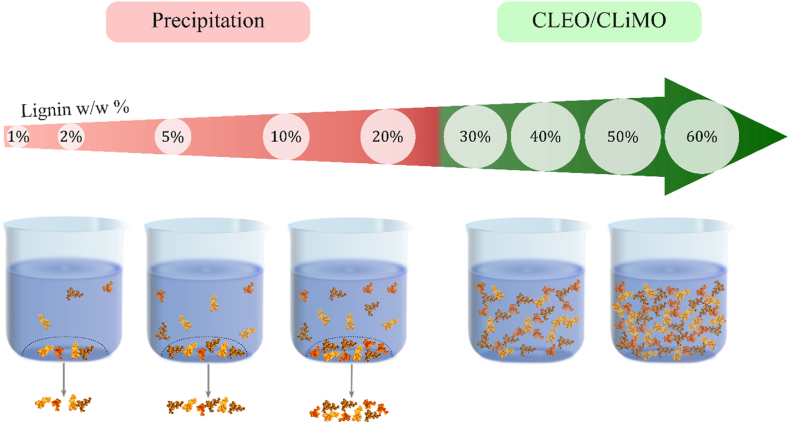
Illustration of lignin stability phenomenon observed for ethanol- and methanol dispersible lignin with lignin concentrations between 1 and 60 wt%. The illustration is based on Supplementary Fig. S1. Lignin concentrations in the stability regime are colored green.

The central property of CLEO/CLiMO fuels is colloidal stability. The lignin concentrations after ethanol and methanol fractionation were 10.2 wt% and 10.6 wt%, respectively. These lignin dispersions were used to produce concentrations down to 1 wt% through dilution, and up to 60 wt% through rotor-evaporation. As illustrated in Fig. 1 and Fig. S1, both ethanol- and methanol-dispersed lignin demonstrated a more pronounced visual precipitation as lignin concentrations increased to 20 wt% and transitioned to stability at ≤30 wt%. This slightly differs from the thermolytic solvolysis lignin, which did not precipitate between 1 wt% and 10 wt%. This might be explained by the approximately 40 % reduction in molecular weight, which was previously observed from thermolytic solvolysis of PB1000 [14]. Consequently, as larger lignin molecules are more likely to agglomerate than smaller molecules [21], the larger lignin molecules in CLEO/CLiMO might explain the apparent precipitation at lower concentrations.

### 3.3 Energy density

**Fig. 2:**
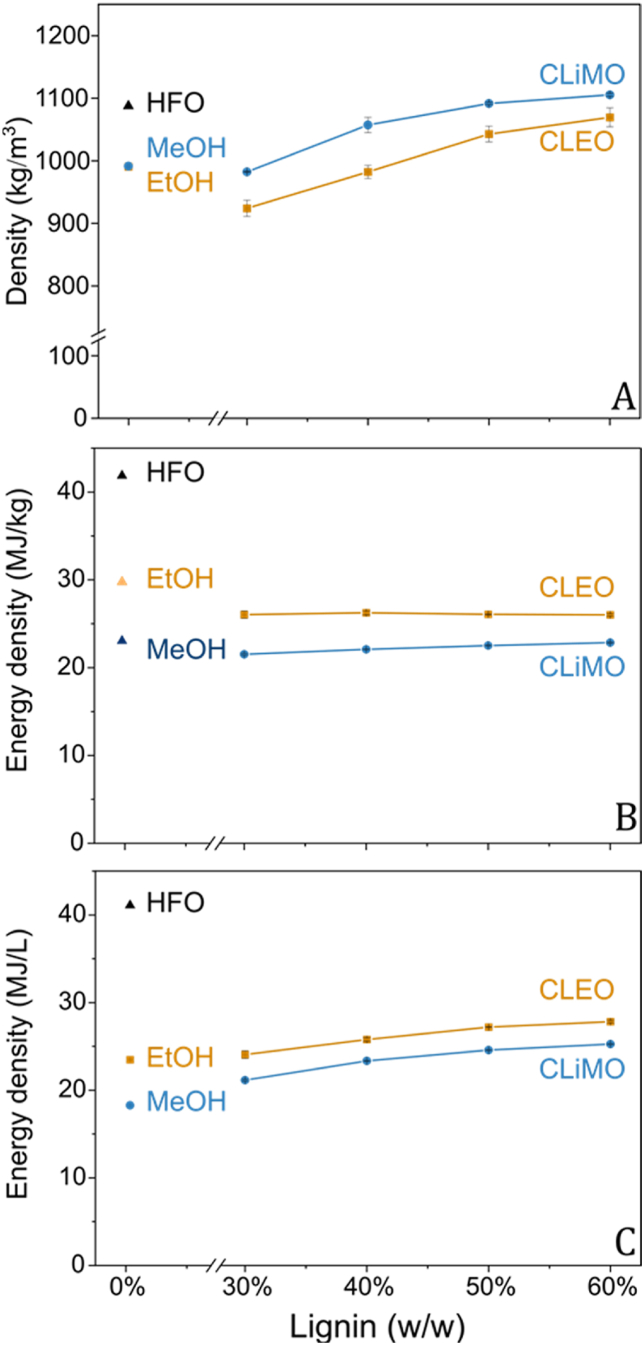
Density and energy density of HFO, 100 % ethanol, 100 % methanol as well as CLEO and CLiMO at lignin concentrations between 30 and 60 wt%. (A) Gravimetric energy density, (B) volumetric energy density and (C) density.

Energy density is a crucial metric for evaluating maritime fuels as it determines how much energy a given volume of fuel can deliver, influencing fuel storage requirements and compatibility with existing ship engine technologies. Fig. 2A illustrates a clear rise in density in response to higher lignin percentages, increasing from ∼800 kg/m^3^ of the pure solvents, to densities exceeding 1000 kg/m^3^ at 60 wt% lignin in both CLEO and CLiMO. As displayed in Fig. 2B, gravimetric energy densities remained relatively even between ∼22 MJ/kg for CLiMO and ∼26 MJ/kg for CLEO across varying lignin concentrations, converging towards the energy density of 25.0 MJ/kg, as measured for PB1000. In examining the volumetric energy densities shown in Fig. 2C, there is a noticeable increase in energy density with higher lignin concentrations in both CLEO and CLiMO. Specifically, CLEO peaks near 27.8 MJ/L, while CLiMO approaches 25.3 MJ/L. Consequently, compared to ethanol and methanol, CLEO and CLiMO demonstrated 19 % and 39 % higher volumetric energy densities, respectively.

Currently, most containerships are equipped with a traditional marine 2-stroke diesel engine with fuel systems incompatible with oxygen- and water-rich biofuels like CLEO and CLiMO [16]. To enable the effective integration of such fuels, retrofitting or adapting existing engines is needed [16]. The recent adoption of methanol fuel highlights the transition towards more sustainable fuels [24]. However, with a volumetric energy density of 18.2 MJ/L, methanol reduces the possible travel distance before refueling by 43 % compared to HFO. Alternatively, methanol-going vessels require much larger fuel tanks, occupying valuable space otherwise used for cargo. By using CLiMO instead of pure methanol, the volumetric energy density would be substantially closer to HFO. Even though CLEO and CLiMO dispersions do not reach the volumetric energy density of HFO, the results demonstrate how adding lignin to alcohol significantly increases the volumetric energy densities, thus reinforcing the potential role of lignin-based fuels as a more sustainable fuel option for the shipping industry.

### 3.4 Ash and elemental analysis

A full elemental analysis was performed, and ash content was determined for CLEO and CLiMO, as dispersed minerals in marine fuels can cause significant abrasion and corrosion within the engine fuel system [25]. Recommended concentrations of specific minerals are listed in the standard shipping fuel specification ISO 8217 2017 [17]. The elemental analysis of lignin fuels demonstrated how alcohol fractionation is also associated with substantial mineral fractionation. Considerably lower mineral content was observed in the dispersed fractions compared to the undispersed fractions (Table S2). However, depending on lignin content, elements like phosphorus, vanadium, and sodium showed varying compliance levels with the ISO 8217, underscoring the need for further mineral reduction strategies.

### 3.5 Viscosity and flow behavior

**Fig. 3:**
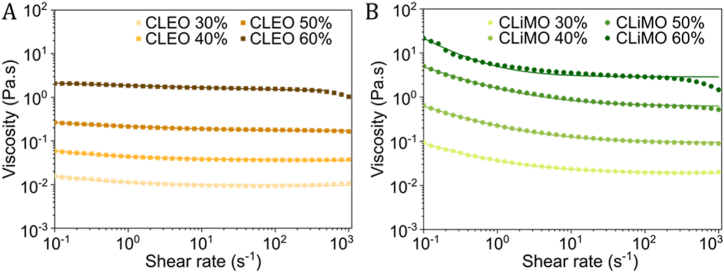
Viscosity of CLEO (A) and CLiMO (B) as a function of shear rate for lignin concentrations ranging from 30 to 60 wt%.

Viscosity influences fuel pumpability and spray atomization, which both are critical for engine performance [26]. Flow curve analysis across shear rates ranging from 10^−1^ and 10^3^ s^−1^ revealed higher viscosities at increased lignin concentrations for both CLEO and CLiMO (Fig. 3A–B). CLiMO clearly exhibited shear-thinning behavior, especially evident for higher lignin concentrations with the lowest flow behavior index of 0.04 for CLiMO 60 % (Table 1). Similarly for both CLEO and CLiMO, lignin concentrations strongly influenced viscosity, exhibiting a 100-fold increase as concentrations rose from 30 wt% to 60 wt%. This finding underscores the significance of maintaining precise control over the lignin concentration to ensure stable fuel properties. At lower shear rates, CLiMO dispersions exhibited an order of magnitude higher viscosity than CLEO dispersions at equivalent lignin concentrations. However, viscosity values converged at higher shear rates due to the higher degree of shear-thinning behavior of CLiMO dispersions.

**Table 1:** Results of Sisko model fitting.

Sample	*η*_*∞*_	*K*	*n*	R^2^
CLEO 30 %	0.009	0.002	0.44	0.95
CLEO 40 %	0.036	0.008	0.54	0.99
CLEO 50 %	0.16	0.05	0.7	0.99
CLEO 60 %	1.02	0.85	0.88	0.98
CLiMO 30 %	0.019	0.017	0.39	0.99
CLiMO 40 %	0.09	0.13	0.39	0.99
CLiMO 50 %	0.62	0.98	0.36	0.99
CLiMO 60 %	2.88	2.08	0.04	0.98

The Sisko model, applied to describe shear-thinning behavior and the constant viscosity regime, yielded fitting parameters listed in Table 1. The consistency index, *K*, reflecting the fluid's internal structure and the strength of intermolecular forces, showed a proportional increase with lignin concentrations. The shear-thinning degree, indicated by the value of *n*, was more pronounced in CLiMO than in CLEO. Notably, CLEO dispersions exhibited increased *n* values at higher lignin concentrations, likely due to the formation of shear-sensitive aggregates inefficiently packed at elevated lignin levels, explaining the flat viscosity profile in the 10^−1^ to 10^−3^ s^−1^ shear range. Conversely, CLiMO dispersions maintained consistent *n* values, except at 60 wt% lignin, where a sharp viscosity decrease was observed at initial shear rates. This behavior might be attributed to lignin in CLiMO forming more aggregated structures that break down at high shear rates, inducing pronounced shear-thinning. Once these bonds effectively break at elevated shear rates, the viscosity variation between CLEO and CLiMO dispersions diminishes. While the observed 100-fold increase in viscosity relates to the increased molecular interactions and colloidal stability of lignin, this characteristic does not pose a challenge if fuel systems are tailored appropriately, similar to how vessels currently handle varying fuel types like HFO and low-sulfur distillates [27].

### 3.6 Small-angle X-ray scattering

**Fig. 4:**
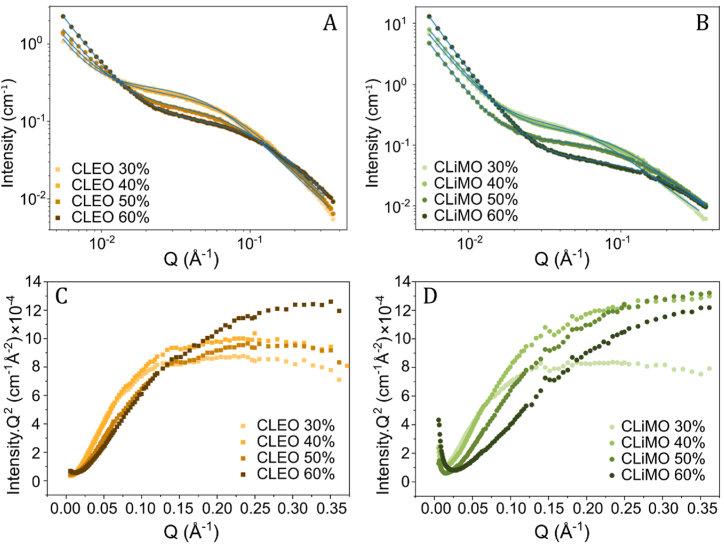
SAXS profiles and corresponding Kratky plots for CLEO (A, C) and CLiMO (B, D) at lignin concentrations from 30 wt% to 60 wt% lignin.

SAXS was employed to assess the impact of lignin concentration and solvent on particle morphology and aggregation behavior. Fig. 4A–B shows the scattering profiles of CLEO and CLiMO dispersions at lignin concentrations between 30 wt% to 60 wt%. The observed upturn at low Q in both CLEO and CLiMO suggests aggregation of larger clusters of lignin molecules. All dispersions showed similar smooth upturn slope at low Q, which suggests the overall large-scale structure of lignin aggregates are similar across lignin concentrations and solvent types, with relatively uniform aggregate sizes. However, CLiMO dispersions demonstrated a more extended upturn compared to CLEO dispersions, indicating a higher degree of aggregation in CLiMO dispersions, which was also observed from the flow curve measurements. The largest differences among lignin concentration and solvent types were observed in mid Q region, corresponding to the size and shape of the individual lignin particles or aggregates. A notable concentration-dependent trend was observed in both CLEO and CLiMO, where higher lignin concentrations corresponded to lower scattering intensities. This might signify a reduction in the contrast between the particles and the medium as the solvent fraction is reduced and the lignin concentration increased. The low Q and high Q data were modeled using a combined power law and Gaussian chain model, as described in supplementary materials. The model fitting suggests that lignin in both types of dispersions forms aggregates of polymer coils, but also that the coil shape and dimensions change gradually by increasing lignin concentrations.

The standard Kratky plots (Intensity × Q^2^ vs. Q) for CLEO and CLiMO dispersions are presented in Fig. 4C–D. These plots are widely used to identify the conformation of the polymeric chains present in the polymeric dispersions [28]. In the Kratky plot, different shapes represent different conformations of the polymeric chains: 1) a bell shape or a distinct peak represents the scattering from a compact globular shape conformation, 2) a pseudo-linear rise in the plot suggests rod-like conformation, and 3) a rise to a plateau suggests a random coil conformation [29,30]. The scattering signature of CLEO dispersions resembles a typical globular random coil shape, as the scattering profile rises to a plateau at high Q values. On the other hand, the CLiMO dispersions scattering signatures only showed a rise to plateau at high Q for 30 wt%, whereas the scattering profile of CLiMO dispersions with higher lignin concentrations continued to grow. Thus, both CLEO and CLiMO have a shift in their scattering profile from plateau to continuous growth, suggesting that the lignin conformation changes from a compressed globular coil to a more elongated unfolded coil as lignin concentration increases.

**Fig. 5:**
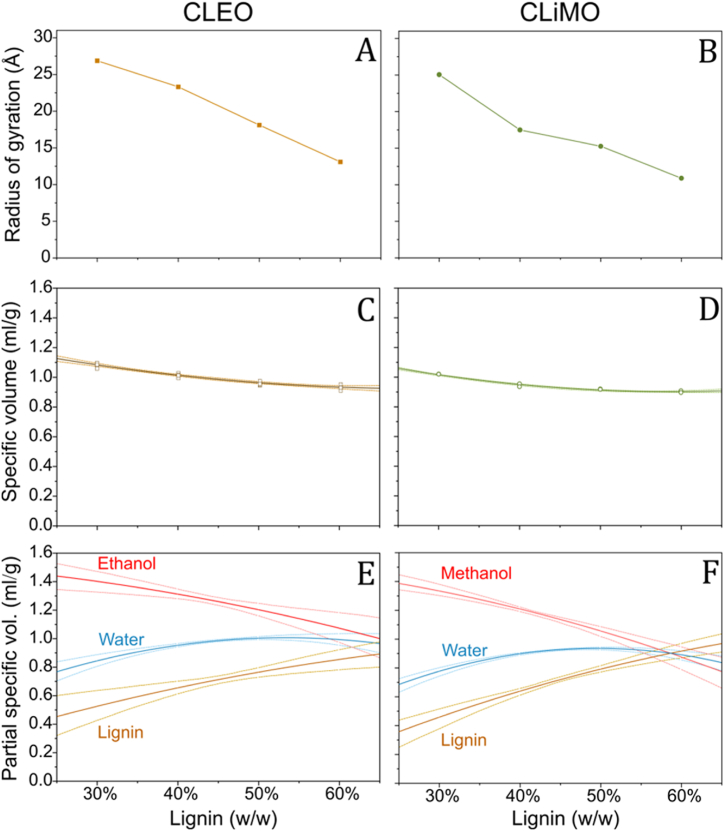
Radius of gyration of lignin primary structure in (A) CLEO and (B) CLiMO at 30 to 60 wt% lignin. Specific volume of (C) CLEO (brown) and (D) CLiMO (green) at 30 to 60 wt% lignin with 95 % confidence bands of second order polynomial fit, squares and circles represent individual measurements. Partial specific volume of alcohol (red), water (blue) and lignin (brown) in (E) CLEO and (F) CLiMO at 30 to 60 wt% lignin with 95 % confidence intervals.

In Fig. 5A–B, the radius of gyration (R_g_) of the primary structure of ethanol- and methanol-dispersed lignin is depicted, showing a wide range of sizes R_g_ ranging from 27 to 11 Å, dependent on lignin concentrations. In both ethanol- and methanol-dispersed lignin, the R_g_ decreased with increasing lignin concentrations. Additionally, the R_g_ of ethanol-dispersed lignin was generally 10–30 % larger than methanol-dispersed lignin. Similar observations have been made on solvolysis lignin, where the difference in R_g_ was related to the smaller size and higher polarity of methanol [15]. This might be explained by the smaller size of methanol, enabling it to take up less space between and within lignin molecules, thus facilitating a slightly tighter packing compared to ethanol.

### 3.7 Partial specific volumes of water, solvent, and lignin

To understand how lignin and solvent volumes vary with different lignin concentrations, we calculated the Partial Specific Volume (PSV) for each component in CLEO and CLiMO dispersions. Our analysis indicated that a second-order polynomial fitting was optimal since third-order polynomials yielded non-significant coefficients. Thus, utilizing the second-order polynomial coefficients, we computed the PSV for each component as detailed in supplementary materials. Remarkably, PSV of lignin molecules exhibited significant variation with concentration changes. Fig. 5C–D illustrate the specific volumes (inverse of density) of CLEO and CLiMO. These curves exhibit a non-linear decrease with increasing lignin concentration, accompanied by a slight upward curvature. The specific volume reduction at increased concentrations primarily results from the trivial effect of the higher density of lignin. On the contrary, the observed non-linearity suggests complex volumetric interactions among the components as the compositions change. For lignin in CLEO, PSV expanded from 0.45 ml/g to 0.89 ml/g, while for lignin in CLiMO, it ranged from 0.36 ml/g to 0.97 ml/g (Fig. 5E–F). Concurrently, the PSV of ethanol and methanol decreased from 1.44 ml/g to 1.00 ml/g and from 1.39 ml/g to 0.88 ml/g, respectively. We found no comparable literature data on high-concentration lignin-alcohol dispersions. However, the PSVs for dilute alkali lignins and lignosulfonates, ranging from 0.61 to 0.74 ml/g, align well with our observations [[31], [32], [33]].

**Fig. 6:**
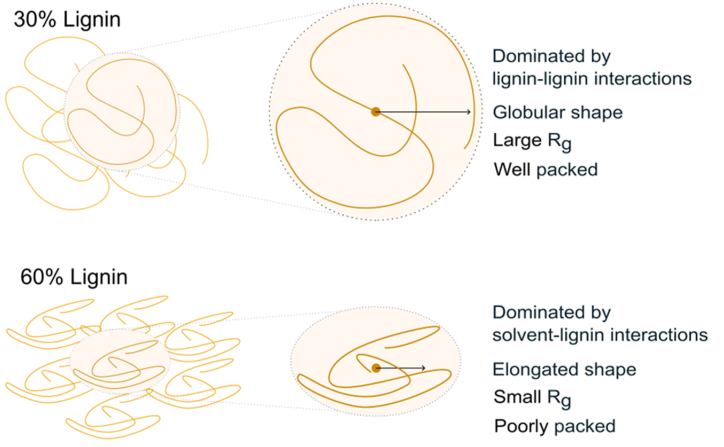
Graphical illustration of hypothesized change in lignin morphology of CLEO and CLiMO going from 30 to 60 wt% lignin. The illustration emphasizes the change in lignin self-interactions and change in radius of gyration, whereas the change in volume is not directly illustrated.

At first glance, the simultaneous decrease in R_g_ and increase in PSV may appear contradictory. The reduction in the R_g_ might be rationalized in terms of the lignin-to-solvent ratio and the composition of the solvent. With constant water content of 10 wt%, increasing lignin concentrations lead to a higher water-to-alcohol ratio in the residual solvent, affecting solvent quality and availability (see Supplementary Materials, Table S3). This transition affects lignin polymer segments to shift from an extended spatial configuration with significant solvent interaction, to a more compact structure, predominantly engaging in self-interaction. This can be seen as a reduction of the spatial extension of the lignin polymers, i.e., a reduction of R_g_. Concurrently, the Kratky plot revealed a transition from globular shapes at low lignin concentrations, to a more elongated shape as a result from the increased lignin-lignin interactions at high lignin concentrations (Fig. 6). The increase in the PSV of lignin can be rationalized by being associated with a less efficient packing of lignin connected to lignin-lignin interactions compared to lignin-solvent interactions. Thus, the PSV can be understood as the volume increases upon adding an infinitesimal extra amount of a specific component of the mixture. Adding more lignin to an already concentrated system leads to more lignin-lignin interactions, resulting in a higher PSV. Thus, adding more alcohol would induce a decrease in the lignin-lignin interactions, consistent with the observation of a low PSV of alcohol, which is much lower than PSV of pure alcohol, 1.27 g/mL for ethanol and 1.26 for methanol, in this concentration range. CLEO and CLiMO present promising prospects for the development of lignin-based fuels. These fuels offer a simpler alternative to conventional lignin oils produced from RCF or thermolytic solvolysis. However, more research is needed to enhance production efficiency and applicability. Future studies could explore the use of alternative solvents, co-solvents and optimizing process parameters. Additionally, reducing energy consumption during solvent evaporation would improve the environmental and economic viability of the production process [12,34]. Furthermore, to ensure the scalability of CLEO and CLiMO production, it is crucial to explore a broader range of lignin sources. This would require evaluation of these alternative lignin sources in terms of their purity, functional properties, and suitability for alcohol fractionation processes to ensure that the quality and performance of CLEO and CLiMO are maintained or enhanced. Transitioning from technical lignins such as PB1000 to lignin residues from second-generation (2G) biorefineries could offer a more cost-effective and sustainable source of lignin [35]. Additionally, CLiMO could be produced from Kraft lignin and methanol generated from Power-to-X technologies, further enhancing the sustainability of lignin-based fuels [36].

## 4 Conclusions

This study demonstrates a straightforward method of alcohol fractionation that produces stable lignin dispersions with concentrations above 30 wt%. These dispersions were associated with the formation of a network of interacting lignin aggregates. As lignin concentration increased, the particles exhibited less efficient packing, characterized by an expanded PSV and a reduced R_g_, which was associated with a 100-fold increase in viscosity when comparing dispersions with 30 wt% and 60 wt% lignin. Notably, these high-concentration lignin dispersions exhibit a 19 %–39 % increase in volumetric energy density compared to their alcoholic solvents, underscoring the potential of CLEO as a promising candidate for lignin-based marine fuel applications.

## CRediT authorship contribution statement

**Tor I. Simonsen:** Writing – original draft, Visualization, Methodology, Investigation, Conceptualization. **Saket Kumar:** Writing – review & editing, Software, Investigation. **Demi T. Djajadi:** Writing – review & editing, Supervision, Conceptualization. **Jacob J.K. Kirkensgaard:** Writing – review & editing, Software. **Jens Risbo:** Writing – review & editing, Methodology, Formal analysis, Conceptualization. **Sune T. Thomsen:** Writing – review & editing, Supervision, Conceptualization. **Yohanna C. Orozco:** Writing – review & editing, Validation, Methodology.

## Data availability

Data supporting this study are available within the article and/or from the corresponding author upon request.

## Funding

This work was supported by 10.13039/501100022591The Energy Technology Development and Demonstration Program [Grant number 64020–1101]. They were not involved in the collection, analysis or interpretation of data. Neither were they involved in the writing of the article nor in the decision to submit the article for publication.

## Declaration of competing interest

The authors declare the following financial interests/personal relationships which may be considered as potential competing interests:Tor Ivan Simonsen reports financial support was provided by 10.13039/501100022591The Energy Technology Development and Demonstration Programme. Yohanna Orozco Cabrera has patent #11306264 issued to A.P. MØLLER—MÆRSK A/S, KØBENHAVNS UNIVERSITET.
