# Supplementary material for: Characterization and structural analysis of alcohol-fractionated lignin biofuels processed at ambient temperature
Source: Heliyon. 2024 Oct 11;10(20):e39249. doi: 10.1016/j.heliyon.2024.e39249 (PMC11532828; doi:10.1016/j.heliyon.2024.e39249)
Supplement: Multimedia component 1 [file mmc1.docx]

Supplementary Materials for:

# Characterization and Structural Analysis of Alcohol-Fractionated Lignin Biofuels Processed at Ambient Temperature

Tor I. Simonsen^a,‡,^^[[1]](#footnote-1)^, Saket Kumar^b,‡^, Demi T. Djajadi^a^, Jacob J. K. Kirkensgaard^c,d^, Jens Risbo^c^, Sune T. Thomsen^a^, and Yohanna C. Orozco^a^

^a^Department of Geosciences and Natural Resource Management, University of Copenhagen, Frederiksberg C, Copenhagen 1958, Denmark

^b^Department of Chemical Engineering, School of Energy Technology, Pandit Deendayal Energy University, Gandhinagar 382007, India

^c^Department of Food Science, University of Copenhagen, Frederiksberg C, Copenhagen 1958, Denmark

# ^d^Niels Bohr Institute, Faculty of Science, University of Copenhagen, Universitetsparken 5, Copenhagen 2100, Denmark

**Elemental analysis**

Elemental analysis was done by inductively coupled plasma-mass spectrometry (ICP-MS). Prior to analysis 100 mg sample was added to 10 ml concentrated HNO_3_ (sub-boiling quality) directly in the microwave vessel. After closing the vessel, a pressurized digestion in microwave oven was done for 45 minutes followed by a dilution 10 times with Milli-Q water.

Determination of Si was done separately and digested in the microwave oven by a two-step procedure prior to analysis. 100 mg dry sample was added 1.8 ml Milli-Q water, 3.2 ml 1.6 M HNO_3_ (sub-boiling quality) and 5 ml 30% H_2_O_2_ directly in the microwave vessel, and pressurized digestion was done for 28 minutes. After cooling, 7.5 ml 1 M NaOH was added followed by a second digestion for 25 min. After digestion, 100 µl 24 M HN_4_F and 750 µl concentrated HNO_3_ were added and subsequently diluted to a final volume of 50 ml with Milli-Q water.

The ICP-MS (iCAP-Q, Thermo) was running in Kinetic energy discrimination (KED) mode using He as collision gas, except for silica, samples which used H_2_ as collision gas. For quantification, external calibration was used, and internal standards were used to compensate drift and matrix effects. The NIST 1515 Apple Leaves reference material was analyzed together with the samples to evaluate the accuracy and precision of the analysis. NIST 1640a and NIST 1643f were used as calibration verification standards.

# **SAXS fitting parameters**

The low Q and high Q data are modeled using a combination of a power law and a Gaussian coil model to fit the low and high Q region, respectively. The combined model for the measured scattering intensity I(Q) is of the form:

$$I\left( Q \right)=Scale\cdot\left( {Scale}_{power}\cdot Q^{-m}+{Scale}_{coil}\cdot I_{0}\cdot P\left( Q \right) \right)+Background$$

With

$$P(Q)={2\left[ \exp\left( -\left( QR_{g} \right)^{2} \right)+\left( QR_{g} \right)^{2}-1 \right]}/{\left( QR_{g} \right)^{4}}$$

where, *I_0_* is zero *Q* intensity, *m* is the power-law exponent and *P(Q)* is the Gaussian coil single-chain form factor. More details on the fitting parameters are found in supplementary materials, Table S1.

**Table S1:** Small-angle x-ray scattering parameters for lignin dispersions, detailing power law and Gaussian coil model fitting results.

| Sample | *I_0_*_, coil_  (cm^-1^) | *R_g_*_, coil_  (Å) | ^a^Scale_power_ | Slope_power_ | Poly-dispersity of R_g_ | $\boldsymbol{\chi}^{2}$ |
| --- | --- | --- | --- | --- | --- | --- |
| CLEO-30% | 0.19 | 26.87 | 9.6e-6 | 3.07 | 0.18 | 14.60 |
| CLEO-40% | 0.17 | 23.31 | 8.06e-7 | 2.71 | 0.19 | 9.20 |
| CLEO-50% | 0.11 | 18.10 | 4.4e-6 | 2.46 | 0.24 | 18.94 |
| CLEO-60% | 0.11 | 13.09 | 3.11e-6 | 2.59 | 0.25 | 12.28 |
| CLiMO-30% | 0.14 | 25.05 | 4.41e-7 | 3.17 | 0.01 | 18.56 |
| CLiMO-40% | 0.14 | 17.50 | 5.14e-7 | 3.18 | 0.01 | 13.49 |
| CLiMO-50% | 0.11 | 15.26 | 7.8e-8 | 3.44 | 0.25 | 4.30 |
| CLiMO-60% | 0.07 | 10.89 | 2.1e-7 | 3.44 | 0.26 | 7.70 |

^a^The overall Scale and Scale-coil were 1.

# **Partial specific volume**

The partial specific volume of a component in a multi-component system is defined as the partial derivative of the volume with respect to the mass of the component under consideration:

$$\bar{v}_{i}=\left( \partial V/\partial m_{i} \right)_{T, p,m_{j}},$$

where index $i$ denotes the component under consideration and index $j$ denotes the other components of the system. By differentiation using the chain rule using the weight fraction of lignin, *w_l_*, as the primary independent variable one can readily obtain expressions for the partial specific volume $\bar{v},$ of each lignin ($l$) and alcohol ($a$):

$$\bar{v}_{l}=\bar{V}+\partial\bar{V}/\partial w_{l}\cdot\left( 1-w_{l} \right)$$

And,

$\bar{v}_{a}=\bar{V}-\partial\bar{V}/\partial w_{l}\cdot\left( w_{l}+w_{\text{H}_{\text{2}}\text{O}} \right)$.

As the water content is constant in the present experiments ($w_{\text{H}_{\text{2}}\text{O}}=0.1)$, the partial specific volume of this component cannot directly be found from the chain rule, but can be found because the partial specific volume adds up to the specific volume through the completeness relation:

$\bar{V}= {w_{l} \bar{v}}_{l}+ {w_{a} \bar{v}}_{a}+w_{\text{H}_{\text{2}}\text{O}}\bar{v}_{\text{H}_{\text{2}}\text{O}}$,

and the missing partial specific volume can be found as:

$$\bar{v}_{\text{H}_{\text{2}}\text{O}}=\left( \bar{V}-{w_{l}\bar{v}}_{l}-w_{a}\bar{v}_{a} \right)/w_{\text{H}_{\text{2}}\text{O}}$$

In more technical terms, data for $\bar{V}$ as function of $w_{l}$ is fitted to a polynomial in order to facilitate making the derivatives. The polynomial with the highest order with statistically significant coefficients was used. The 95 % confidence intervals of the polynomial estimates of $\bar{V}, \bar{v}_{l}{, \bar{v}}_{a}$and $\bar{v}_{\text{H}_{\text{2}}\text{O}}$ was assessed by a bootstrapping procedure.


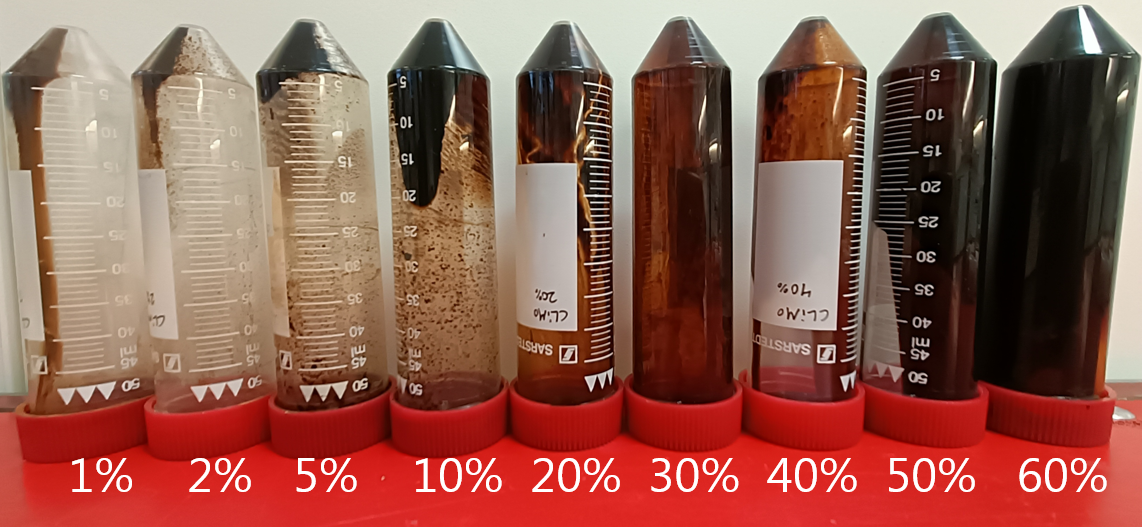

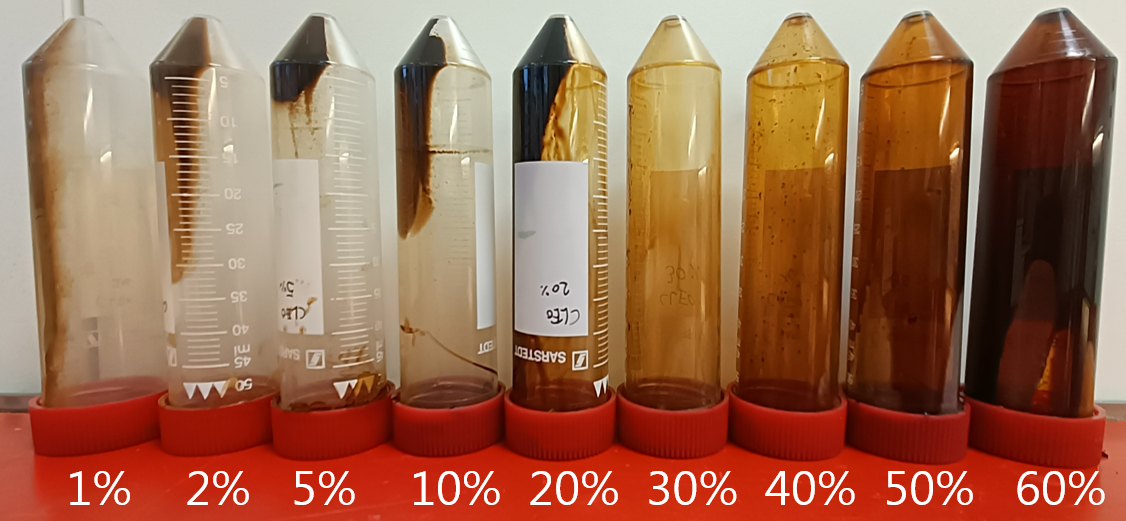


A

B

**Figure S1**. Concentration dependent lignin precipitation. Photograph of lignin precipitation in ethanol (A) and methanol (B) at varying lignin loadings. Concentrations were from left to right: 1 wt%, 2 wt%, 5 wt%, 10 wt%, 20 wt%, 30 wt%, 40 wt%, 50 wt% and 60 wt%.

**Table S2.** Elemental analysis and ash content of lignin fractions in w/w. Standard deviations are given in brackets. *RMK700 is marine residual fuel standards defined by ISO8217 2017.

| **Sample** | **Ash** | **S** | **V** | **Si + Al** | **Zn** | **P** | **Na** | **Ca** |
| --- | --- | --- | --- | --- | --- | --- | --- | --- |
|  | (%) | (ppm) | (ppm) | (ppm) | (ppm) | (ppm) | (ppm) | (ppm) |
| RMK 700* | 0.15 | 5000 | 600 | 80 | 15 | 15 | 100 | 30 |
| CLEO 30% | 0.09 (0.01) | 1689 (5.4) | 0.2 (0.00) | 37.6 (1.5) | 1.0 (0.0) | 9.9 (0.4) | 310 (3) | 5.2 (1.8) |
| CLEO 60% | 0.19 (0.02) | 3379 (10.8) | 0.5 (0.00) | 75.2 (3.0) | 2.0 (0.0) | 19.8 (0.7) | 619 (6) | 10.3 (3.5) |
| CLiMO 30% | 0.17 (0.01) | 2024 (36.1) | 0.4 (0.00) | 45 (3.8) | 1.1 (0.2) | 11.7 (0.2) | 701 (5) | 5.7 (0.6) |
| CLiMO 60% | 0.34 (0.02) | 4048 (72.2) | 0.8 (0.00) | 90 (7.6) | 2.1 (0.3) | 23.5 (0.4) | 1402 (10) | 11.4 (1.2) |
| EtOH-dispersed | 0.3 (0.04) | 5631 (18) | 0.8 (0.00) | 125 (5.0) | 3.3 (0.0) | 32.9 (1.2) | 1032 (10) | 17.2 (5.9) |
| MeOH-dispersed | 0.6 (0.03) | 6747 (120) | 1.3 (0.00) | 150 (12.7) | 3.6 (0.5) | 39.1 (0.7) | 2336 (17) | 19.1 (2.0) |
| EtOH-undispersed | 2.92 (0.03) | 12310 (220) | 3.8 (0.00) | 2669 (66.4) | 8.5 (0.5) | 42.4 (0.9) | 8974 (106) | 277.8 (7.9) |
| MeOH-undispersed | 3.09 (0.12) | 11485 (105) | 3.6 (0.00) | 2135 (133) | 6.7 (0.2) | 43.7 (1.0) | 8005 (77) | 234.0 (1.3) |
| PB1000 | 1.6 (0.23) | 9624 (159) | 2.4 (0.00) | 1532 (39.7) | 4.7 (0.3) | 44.3 (0.7) | 6305 (124) | 154 (6.2) |

**Table S3.** Solvent composition at different lignin concentrations. Composition of CLEO and CLiMO dispersions given in wt% and their relative water concentration given in wt% of total solvent.

|  | CLEO/CLiMO 30% | CLEO/CLiMO 40% | CLEO/CLiMO 50% | CLEO/CLiMO 60% |
| --- | --- | --- | --- | --- |
| Lignin | 30% | 40% | 50% | 60% |
| Alcohol | 60% | 50% | 40% | 30% |
| Water | 10% | 10% | 10% | 10% |
| Water% of solvent | 14% | 17% | 20% | 25% |

1. Corresponding author. Email: [tsi@ign.ku.dk](mailto:tsi@ign.ku.dk), Phone: +45 28561408.
   ‡These authors contributed equally. [↑](#footnote-ref-1)
